# Research progress on traditional Chinese medicine compounds in autoimmune-related skin diseases

**DOI:** 10.3389/fimmu.2025.1629288

**Published:** 2025-10-02

**Authors:** Yuhan Wang, Pengzhi Bu, Yu Deng, Wenyingzi Zhao, Guangtao Pan

**Affiliations:** ^1^ Hubei University of Chinese Medicine, Wuhan, Hubei, China; ^2^ Chengdu University of Traditional Chinese Medicine, Chengdu, Sichuan, China; ^3^ School of Chinese Medicine, Macau University of Science and Technology, Macao, Macao SAR, China; ^4^ Wuxi Affiliated Hospital of Nanjing University of Chinese Medicine, Wuxi, Jiangsu, China; ^5^ Yancheng Hospital of Traditional Chinese Medicine Affiliated to Nanjing University of Chinese Medicine, Yancheng Hospital of Traditional Chinese Medicine, Yancheng, Jiangsu, China

**Keywords:** autoimmune diseases, traditional Chinese medicine, psoriasis, atopic dermatitis, vitiligo, Sjögren’s syndrome

## Abstract

**Background:**

Autoimmune-related skin diseases such as psoriasis, atopic dermatitis (AD), vitiligo, and Sjögren’s syndrome (SjS) are characterized by chronic inflammation, immune dysregulation, and complex pathogenesis. Current therapeutic options are limited by side effects, poor efficacy, and high costs. Traditional Chinese medicine (TCM) compounds have demonstrated promising immunomodulatory, anti-inflammatory, and antioxidant effects, offering a novel alternative for managing these conditions.

**Objective:**

To systematically review the pharmacological activities and therapeutic potential of bioactive compounds derived from traditional Chinese medicine in treating autoimmune-related skin diseases.

**Methods:**

A comprehensive literature review was conducted, focusing on studies that investigated the efficacy, mechanism, and delivery strategies of TCM-derived compounds. Diseases covered include psoriasis, AD, vitiligo, and SjS. Mechanistic pathways such as JAK/STAT, PI3K/AKT, and NF-κB were evaluated alongside compound-specific delivery systems.

**Results:**

Numerous TCM compounds, including curcumin, resveratrol, baicalein, berberine, ginsenosides, and paeoniflorin, exhibited therapeutic effects in preclinical and clinical models. These effects were mediated through various pathways, including inhibition of pro-inflammatory cytokines, restoration of skin barrier function, and modulation of immune cell balance. Nano-based delivery systems further enhanced the stability and bioavailability of these compounds. The compounds also showed potential in reducing oxidative stress and improving melanocyte survival in vitiligo, or restoring glandular secretion in SjS.

**Conclusion:**

TCM compounds present a valuable resource for the development of safer and more effective therapies for autoimmune-related skin diseases. Further studies are warranted to clarify their mechanisms, optimize delivery systems, and validate clinical efficacy. These natural compounds represent a promising direction for future dermatological drug discovery.

## Graphical Abstract

This review highlights the mechanism of action and research progress of various Chinese medicine compounds in autoimmune-related skin diseases such as psoriasis, atopic dermatitis (AD), vitiligo, and Sjögren’s syndrome (SjS).

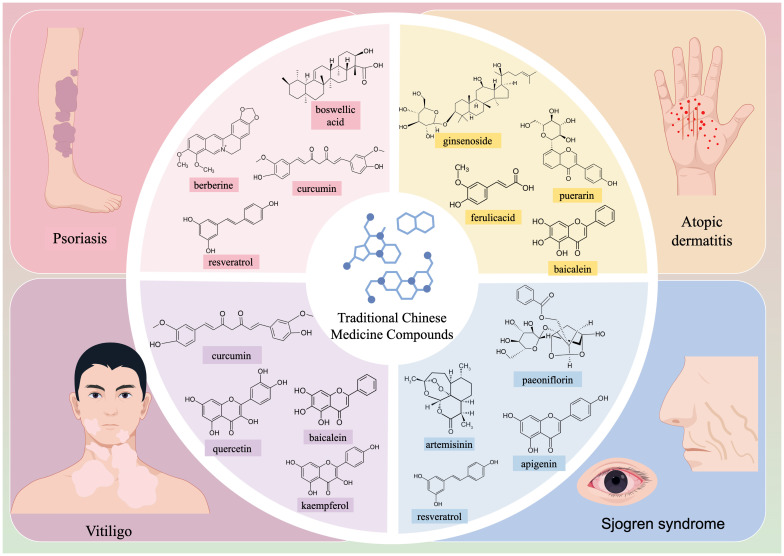

## Background

Psoriasis, atopic dermatitis (AD), vitiligo, Sjögren’s syndrome (SjS), systemic lupus erythematosus (SLE), systemic sclerosis (SSc), and bullous dermatosis (BD) are robustly associated with disruptions in immune homeostasis, and their development is regulated by multiple immune signaling pathways. In recent years, bioactive compounds derived from traditional Chinese herbs have demonstrated promising potential in the treatment of autoimmune-related skin diseases due to their wide availability, diverse molecular targets, and substantial potential for further research and development (1). The keywords used in the literature search are: Autoimmune diseases; Traditional Chinese medicine; Psoriasis; Atopic dermatitis; Vitiligo; Sjögren’s syndrome. The majority of the cited literature consists of papers published in the last 10 years, thereby reporting the latest findings and important conclusions in this research field. We strictly adhered to the principles of systematicity, transparency, and reproducibility during the literature screening process, which is summarized as follows. Based on the research objective of elucidating the mechanisms of action and research potential of various traditional Chinese medicine compounds in autoimmune-related skin diseases, we established strict criteria for literature inclusion, including clearly defined research keywords, determination of research types, and limitation of the time frame. It separately searched for basic and clinical studies related to each skin disease using traditional Chinese medicine compounds. We also excluded duplicate literature and those for which full texts and specific data could not be obtained, and manually applied Boolean logic (MeSH) to combine subject terms and free terms. Furthermore, we established an independent double-blind review mechanism. For literature where the opinions of the two reviewers were inconsistent, a third senior researcher will arbitrate to determine whether to include it. More importantly, it was discussed the consistency and continuity of conclusions in the literature from different periods, as well as points of contention among different studies. This approach aimed to minimize subjective bias and ensure the representativeness and reliability of the cited literature. This review focuses on the research progress and mechanistic understanding of various traditional Chinese medicine (TCM) compounds in autoimmune-related skin diseases. The objective is to provide new therapeutic strategies for these conditions and suggest new directions for the development of drugs targeting autoimmune-related skin diseases.

## Therapeutic potential of TCM compounds in psoriasis

Psoriasis is a common chronic inflammatory skin disease and an autoimmune-mediated genetic disorder. It is characterized by clearly demarcated erythematous plaques covered with white scales, which frequently affect the skin and joints. Conventional treatment options include topical corticosteroids, vitamin D analogs, phototherapy, and systemic oral agents, such as methotrexate and cyclosporine (2). Despite their widespread use, these therapies present notable limitations. Many patients respond poorly to conventional treatment regimens. Moreover, long-term exposure to ultraviolet radiation raises the risk of skin cancer, and systemic medications, such as methotrexate and cyclosporine, can lead to nephrotoxicity, bone marrow suppression, and hepatotoxicity (3). In recent years, increasing exploration of the pathogenesis of psoriasis has brought substantial attention to the use of biologics. Despite this interest, biologics remain costly, and their long-term use has gradually revealed side effects, such as immune dysregulation and potential carcinogenic risks. In contrast, bioactive compounds derived from natural TCMs offer enhanced therapeutic efficacy with fewer adverse effects, making them a promising alternative in psoriasis therapy (4, 5).

### Anti-inflammatory properties of curcumin in psoriasis

CUR, also referred to as turmeric, is a diketone compound derived from the rhizomes of *Zingiberaceae* plants, such as *Curcumae Longa*, *Curcuma phaeocaulis*, *Brassica juncea*, *Murraya koenigii*, *Curcuma aromatica.* It exhibits a wide range of pharmacological activities, including lipid-lowering, anti-tumor, anti-inflammatory, choleretic, and antioxidant properties. Kang et al. (6) clarified the therapeutic potential of CUR for psoriasis in a mouse model. The findings uncovered that CUR markedly suppressed inflammatory cytokine secretion in T cells *in vitro* and affected key pathological indicators in mice, such as ear swelling, body weight, skin thickness, and lymph node weight. Similarly, Cai et al. (7) demonstrated that CUR prominently alleviated the characteristic skin lesions in a psoriatic mouse model, and this effect was robustly associated with the modulation of the IL-6/STAT3 signaling pathway. Given these findings, CUR holds promise as a potential treatment for psoriasis in the future. Biomedical engineers have explored nanocarrier systems for CUR delivery to enhance its solubility and stability, thereby facilitating its therapeutic efficacy as a natural compound-based therapy. Zhang et al. (8) developed a hyaluronic acid-modified ethosomal CUR delivery system and compared its efficacy with conventional ethosomes. This system was designed to target the overexpression of CD44 protein in psoriasis. The findings revealed that the hyaluronic acid-ethosome group notably diminished inflammation and enhanced the accumulation of other anti-psoriatic drugs. Interestingly, Chen et al. (9) formulated a liposomal gel incorporating CUR and retinoic acid, which substantially fostered the solubility and stability of both compounds. Lu et al. (10) developed a curcumin-based ionic liquid hydrogel loaded with ilomastat for topical treatment of psoriasis. Their research delineated that topical application of this hydrogel markedly reduced the expression of inflammatory cytokines, matrix metalloproteinase-8, and collagen-I, leading to significant improvements in psoriatic lesions in a mouse model. Gomez et al. (11) evaluated the combination of CH/alginate NPs loaded with CUR (Cur-CS/Alg NPs) and blue light-emitting diode (LED) light irradiation in suppressing TNF-α-induced psoriatic activity *in vitro*. Human HaCaT keratinocytes were cultured and incubated with TNF-α. Free CUR dissolved in dimethyl sulfoxide (DMSO) and Cur-CS/Alg NPs were added to the cells at final CUR concentrations of 0.05 and 0.1 µg/mL, while DMSO and CS/Alg NPs were used as controls. After treatment, analyzes showed that CS/Alg NPs were not toxic to normal HaCaT cells, while 0.05 µg/mL and 0.1 µg/mL of free CUR and Cur-CS/Alg NPs inhibited hyperproliferation of HaCaT cells with psoriatic inflammation induced by TNF-α.

### Potential of boswellic acid in alleviating psoriatic lesions

Boswellic acid, a bioactive compound extracted from *Olibanum*, exhibits various pharmacological properties, including antioxidant, anti-inflammatory, anti-bacterial, and anti-tumor effects. For example, Togni et al. (12) conducted a comparative study between a formulation containing *Boswellia serrata* resin extract and a placebo. The findings unveiled that the *Boswellia serrata* resin-based formulation notably reduced itching and erythema, highlighting its potential as a promising therapeutic option for psoriasis. Wang et al. (13) provided further evidence that acetyl-11-keto-β-boswellic acid (AKBA) markedly alleviated imiquimod-induced psoriasis-like symptoms in mouse models. This effect was likely achieved through the inhibition of the maturation and differentiation of dendritic cells by suppressing the TLR8 and IRF signaling pathways. Based on these observations, Lou et al. (14) chemically modified AKBA to develop 3-O-cyclohexanecarbonyl-11-keto-β-boswellic acid (CKBA) ointment, which was tested in psoriasis-afflicted mice through topical and systemic administration. The results demonstrated that CKBA notably curbed Th17 cell differentiation and safely reduced psoriasis-like skin inflammation. Lou et al. (15) provided evidence for CKBA as a novel compound that is a promising candidate for further development as a drug for external use to treat mild-to-moderate plaque psoriasis. their data pave the way for the initiation of a Phase 1 clinical trial of the safety profiles of 0.5% and higher doses of CKBA ointments in healthy volunteers.

### Role of resveratrol in psoriasis symptom alleviation

RES, a non-flavonoid polyphenolic compound, is predominantly extracted from *Reynoutria japonica* and *Vitis vinifera*. It has been well-recognized for free radical scavenging and antioxidant properties. RES has been widely applied in dermatological treatments due to its prominent bioactive potential. Notably, Kjær et al. (16) demonstrated that RES could mitigate psoriasis symptoms by modulating the expression of retinoic acid-responsive genes and the interleukin (IL)-17 signaling pathway, particularly by regulating IL-17A and IL-19 mRNA levels. Moreover, Cheng et al. (17) further supported the anti-psoriatic activity of RES when administered topically. In addition to these effects, several studies have explored drug delivery strategies for RES. For example, Khatoon et al. (18) formulated a combination of three natural compounds (CUR, RES, and thymoquinone) with anti-psoriatic activities and demonstrated that the mixture could ameliorate psoriasis-like symptoms in a Balb/c mouse model. Similarly, Elgewelly et al. (19) focused on developing a safe and effective new treatment for psoriasis. In their study, they utilized vesicular elastic nanocarrier gel for RES delivery in the treatment of psoriasis and formulated RES-loaded spanlastics (F1-F12). After screening, they selected two optimal formulations (F10 and F11) and incorporated them into a carbolpol934 gel (G5). The results uncovered significant improvements in reducing erythema and scaling, thereby maximizing the topical anti-psoriatic effects of RES. New advancements have been made in understanding the mechanistic basis of RES in psoriasis therapies. Wang et al. (20) pointed out that RES mitigated imiquimod-stimulated psoriasis-like symptoms in mice by curtailing macrophage infiltration and reducing glycolysis. The use of resveratrol in topical treatment is promising because it has been found to be easily absorbed through the skin. The stratum corneum (SC) is resistant to topical medications, the ideal therapeutic agents should be characterized by low molecular weight (<500 g/mol), moderate lipophilicity (partition coefficient log P 1/4 1–3), and adequate solubility in both water and oil, as well as have a low melting point (21). These properties facilitate effective drug delivery through the skin. Thus allowing resveratrol to better treat psoriasis.

### Berberine targeting psoriasis via the JAK-STAT pathway

BBR is a quaternary ammonium isoquinoline alkaloid isolated from the TCM *Coptidis Rhizoma*. It can also be extracted from such plants as *Coptis chinensis*, *Phellodendron amurense*, and *Berberis diaphana*. In further research, Nimisha et al. (22) developed a nanocapsule gel loaded with *Berberis aristata* extract and compared its efficacy to conventional gel formulations. Their findings revealed that, when applied topically, transferrin-loaded gel notably reduced epidermal thickness and rete ridge length in animal models of psoriasis. Furthermore, Freag et al. (23) developed highly permeable liquid crystalline nanoparticles (LCNPs) loaded with a solubility-enhanced BBR-oleate (OL) complex for psoriasis treatment. When compared to crude BBR, BBR-OL-LCNPs demonstrated a threefold increase in drug accumulation and approximately a tenfold improvement in skin penetration in rat models. These findings highlight the importance of formulating BBR-OL in LCNPs for controlled drug release, retention, and skin penetration in psoriasis therapy. Recent advancements have further elucidated the mechanistic underpinnings of BBR. Sun et al. (24) conducted their study based on previous findings that CDC6 was highly expressed in the epidermal cells of psoriasis patients and that its regulation was associated with the IL-22/STAT3 signaling pathway in keratinocytes. Their study found that BBR restricted CDC6 expression and proliferation in human keratinocytes by disrupting the JAK/STAT3 signaling pathway, suggesting that BBR may serve as a potential therapeutic option for patients with psoriasis. Ma et al. (25) retrieved data from multiple databases and hypothesized that epidermal growth factor receptor (EGFR) could be a critical target in psoriasis treatment. Their study identified that the active compounds in the *Scutellariae Radix*–*Phellodendri Chinensis Cortex* herbal combination, including baicalin and BBR, exhibited strong binding affinity to the EGFR protein. These findings provide initial evidence that the therapeutic effects of the *Scutellariae Radix*–*Phellodendri Chinensis Cortex* herbal combination in psoriasis may be driven by a multi-component, multi-target, and multi-pathway mechanism, thus offering preliminary insights for further exploration of its potential mechanisms of action.

## Mechanisms and role of TCM compounds in AD

AD is a genetically predisposed allergic skin disorder marked by chronic inflammation, recurrence, itching, and inflammation. It is a globally widespread condition, impacting individuals across various age groups, from infancy through adulthood (26). The pathogenesis of AD is strongly linked to immune dysregulation, where Th2 and Th17 immune imbalances are essential in the acute and chronic phases of the disease, respectively (27). With the increasing understanding of AD mechanisms, available treatment options have expanded. Emollients and topical treatments, including corticosteroids and calcineurin inhibitors, are typically prescribed for patients with mild AD. Systemic treatments for moderate to severe AD, such as corticosteroids, cyclosporine, methotrexate, and azathioprine, are designed to target key inflammatory cytokines, such as IL-4, IL-13, and IL-17, which are produced by Th2 and Th17 cells. Despite their effectiveness in managing inflammation, the long-term use of these therapies has shown limited efficacy, with significant concerns regarding contraindications and safety risks (28). In recent years, phototherapy has emerged as a valuable option for the treatment of AD (29). However, frequent or improper use of phototherapy carries risks, such as skin cancer and premature skin aging (30). Current research has demonstrated that several natural compounds can reverse the pathological changes associated with AD. Their potential pharmacological effects provide notable advantages in treating AD, particularly for individuals who are not suitable for conventional medications or phototherapy. With ongoing research into the role of TCM compounds in AD, these compounds are increasingly recognized as potential alternatives for effective AD management.

### Ginsenosides for immune modulation in AD

GSs, also referred to as triterpene saponins, are steroidal compounds predominantly found in medicinal plants of the *Panax* genus. GSs have been recognized as the active ingredients in *Panax ginseng*, and various types of GSs have been isolated, including Rh1, Rh2, Rg3, Rg5, and Rk1. For instance, Zheng et al. (31) delineated that GS Rh1 attenuated the increase in serum IL-6 and total IgE levels while prominently upregulating the expression of IFN-γ and Foxp3 in drainage fluid. This anti-inflammatory effect is achieved by halting histamine release from mast cells and diminishing inflammatory cell infiltration. Similarly, Yin et al. (32) supported that GS Rh2 constrained TSLP expression and curbed the differentiation of naïve CD4^+^ T cells into Th2 cells, thereby reducing inflammation by suppressing pro-inflammatory cytokines at both the protein and transcriptional levels. Sungeun et al. (33) pretreated human keratinocytes (HaCaT cells) with GS Rg5:Rk1 and observed a dose-responsive reduction in TNF-α/IFN-γ-induced TARC/CCL17 expression. Moreover, Rg5:Rk1 lowered the production of nitric oxide (NO) and reactive oxygen species (ROS) in lipopolysaccharide (LPS)-challenged macrophages (RAW 264.7 cells) and diminished the mRNA expression of AD mediators. These observations imply the anti-atopic dermatitis impacts of Rg5 by repressing the NF-κB/p38 MAPK/STAT1 signaling pathway.

### Ferulic acid for skin protection and inflammation reduction

FA, a hydroxycinnamic acid and organic compound, is a phenolic compound widely found in the cell walls of various plants. It is particularly abundant in the seeds of coffee, apple, and artichoke, as well as in the seeds and cell walls of *Oryza sativa*, *Triticum aestivum*, and *Avena sativa*. FA is widely used as a key ingredient in pharmaceuticals for treating cardiovascular diseases and leukopenia. Furthermore, it contributes to skin health and protection, offering aesthetic benefits to humans. The study of Liu et al. (34) proposed that the antidepressant-like effects of ferulic acid in the CUMS-induced depressive-like model. Four weeks administration of ferulic acid not only increased sucrose preference, but also decreased immobility time in mice induced by CUMS. FA could counteract the upregulation of pro-inflammatory cytokines, such asIL-1β, IL-6, TNF-α, and NF-κB caused by chronic unpredictable mild stress. In addition, Zhou et al. (35) demonstrated, using the human monocyte model THP-1 cells, that FA markedly retarded 2,4-dinitrochlorobenzene (DNCB)-stimulated Th2 and IgE responses in peripheral blood. FA dose-responsively restricted DNCB-driven upregulation of CD54 and CD86 on cell surfaces, the secretion of IL-6 and TNF-α, and the activation of NF-κB signaling. These findings highlight the strong anti-inflammatory effects of FA targeting IgE and Type 2 immune responses, suggesting its potential as a therapeutic agent for AD.

### Baicalein for restoring skin barrier and immune balance

BAI, a flavonoid compound, is one of the primary active ingredients in the TCM *Scutellariae Radix*. This compound has been recognized to stimulate apoptosis in cancer cells without harming normal cells and exhibit potent antioxidant activity. BAI has shown promising therapeutic potential in the treatment of AD. In an animal study, Yun et al. (36) illuminated the therapeutic potential of BAI to mitigate AD-like symptoms. Treatment with BAI-harboring hydrogel notably diminished immune cell infiltration in the skin of AD model mice and lowered serum levels of the inflammatory cytokines TNF-α and IL-6. Meanwhile, Huong et al. (37) examined the impact of the combination of arctigenin, hederagenin, and BAI on AD-like skin lesions and elucidated the underlying mechanistic actions. In their study, a 0.5% mixture of arctigenin, hederagenin, and BAI was topically applied to the dorsal skin lesions of DNCB-induced AD model mice, leading to a pronounced alleviation in AD-like symptoms. The histological analysis uncovered that the mixture impeded the inflammatory cytokine production in keratinocytes (HaCaT cells) through the ERK and JNK MAPK signaling pathways and blocked the inflammatory factor secretion in corticotropin-releasing hormone/substance P-stimulated human mast cells (HMC-1 cells). These findings provide preliminary support for the combination of arctigenin, hederagenin, and BAI as a potential candidate for AD therapy.

### Effect of puerarin on inflammatory pathways in AD

PUR, an isoflavonoid compound derived from *Pueraria lobata*, has been delineated to impart several pharmacological effects, including dilating coronary and cerebral arteries, lowering blood pressure, slowing heart rate, and decreasing myocardial oxygen consumption. It can alleviate symptoms such as dizziness, headaches, and neck stiffness in patients with hypertension or coronary artery disease. Additionally, PUR has been reported to restore and enhance memory function, which further broadens its pharmacological profile. In the mechanistic study of Li et al., PUR curbed pro-inflammatory cytokine levels and exerted therapeutic effects on skin inflammation by regulating levels of IL-5, TNF-α, and IL-6. Moreover, PUR blunted the activation of several key signaling pathways, including Akt, STAT-1, and NF-κB, and constrained the expression of chemokines such as CCL-2/5/17/8/10/11. Although PUR shows promise as an alternative therapeutic option for AD, its exact molecular mechanisms and precise targets still require further investigation (38).

## Insights into the use of TCM compounds in vitiligo

Vitiligo is a prevalent acquired depigmentation disorder of the skin and mucous membranes. It can occur on any part of the body and at any age, with a higher incidence in individuals with darker skin tones compared to those with lighter skin. Recent research has firmly established vitiligo as an autoimmune disorder, where melanocytes are particularly vulnerable to oxidative stress. Oxidative stress activates autoreactive cytotoxic CD8^+^ T cells and contributes to the production of IFN-γ, which induces surrounding keratinocytes to secrete chemokines. These chemokines then recruit additional T cells to the skin in a positive feedback loop, thereby accelerating disease progression. CD8^+^ tissue-resident memory T cells have been recognized as key players in the long-term and potential recurrence of vitiligo (39). Current clinical treatments for vitiligo primarily focus on enhancing melanocyte regeneration. Common therapeutic approaches include pharmacotherapy, phototherapy, transplantation, and depigmentation therapies. Corticosteroids and calcineurin inhibitors are the mainstays of treatment, along with superoxide dismutase/pseudocatalase creams, 5-fluorouracil, and JAK inhibitors. Nonetheless, the efficacy of these treatments is often limited, and concerns about the safety of long-term use persist (40). To further curb autoimmunity and facilitate pigmentation, medication therapies are often combined with phototherapy, such as psoralen and ultraviolet A and narrowband ultraviolet B (nbUVB). However, these treatments frequently yield suboptimal results. Prolonged use of medium- to high-potency corticosteroids can cause hyperpigmentation and skin atrophy, and some patients exhibit dismal responsiveness and adherence to calcineurin inhibitors. Phototherapy poses a risk of skin cancer, with a recurrence rate of up to 40% within a year (41). Given these challenges, the search for safer and more effective active compounds for vitiligo treatment is becoming increasingly important. Active compounds extracted from TCM may offer a promising avenue for future research in the development of vitiligo therapies.

### Quercetin for melanocyte protection against oxidative stress

QUE, a flavonoid compound, is commonly extracted from plants such as *Ruta graveolens*, *Phyllanthus emblica*, and *Hypericum monogynum*, especially in the form of glycosides in flowers, leaves, and fruits. As an anti-apoptotic and antioxidant compound, QUE confers protective effects against various forms of organ damage and exhibits anti-inflammatory, anti-bacterial, anti-viral, immunomodulatory, and cardiovascular protective properties. For example, Guan et al. (42) confirmed that QUE fostered melanocyte activity, markedly reduced H_2_O_2_-induced endoplasmic reticulum expansion and dysfunction, and preserved tyrosinase output from the endoplasmic reticulum in melanocytes. This finding highlights the robust potential of QUE in vitiligo treatment. Further research has combined QUE with other compounds in the context of vitiligo studies. For instance, Xu et al. (43) developed a TCM formula containing QUE, luteolin (LUT), and kaempferol (KF) for vitiligo treatment. Based on network pharmacology, they pinpointed the key active ingredients and examined the core targets and signaling pathways involved in Baishi Tablets (BSTs; Chinese herbal compound therapy) for vitiligo. Their study unveiled that QUE, LUT, and KF protected immortalized human melanocyte cell line (PIG1) by blunting oxidative stress and lowering intracellular ROS generation. This investigation elucidates the underlying mechanism of BST therapy and offers new perspectives for vitiligo treatment.

### CUR for skin pigmentation restoration

CUR, a diketone compound extracted from the rhizomes of plants in the *Zingiberaceae* and *Araceae* families, has been widely applied as a natural pigment in the food industry and has gained considerable recognition for its broad clinical applications. In a comparative study, Pravit et al. (44) examined the effects of tetrahydrocurcumin (THC) cream combined with targeted nbUVB phototherapy versus nbUVB monotherapy in inducing repigmentation in vitiligo. Their findings indicated that the combination of THC cream with nbUVB was more effective than nbUVB alone, thus offering support for the potential use of CUR in vitiligo treatment. Ashrafizadeh et al. (45) suggested that CUR activated the Nrf2 signaling pathway through multiple mechanisms to bolster the expression of antioxidant and detoxification-related genes and protect cells from oxidative stress. In addition, Skyvalidas et al. (46) pointed out that highlighted the therapeutic potential of CUR in vitiligo by demonstrating its ability to restrict several pro-inflammatory factors involved in the disease’s progression. Nevertheless, the existing evidence regarding the use of CUR for the treatment of vitiligo remains controversial. For instance, Tu et al. (47) observed that CUR markedly attenuated melanin production in normal human melanocytes. This finding conflicts with conclusions supporting its therapeutic role in vitiligo. Therefore, further clinical or basic research is needed to provide additional clinical support for the role of CUR in vitiligo therapy.

### Benefits of BAI in supporting melanocyte function

BAI, the main flavonoid compound in *Scutellariae Radix*, has long been used in TCM for vitiligo treatment. Liu et al. (48) examined the protective properties of BAI on H_2_O_2_-stimulated apoptosis in human melanocytes. Their findings substantiated that BAI blocked mitochondrion-dependent caspase activation and the p38 MAPK pathway, thereby preventing melanocyte loss. Based on earlier findings, Ma et al. (49) further demonstrated that BAI augmented O-1 expression through the upregulation of the Nrf2 signaling pathway, thereby restoring mitochondrial function and protecting vitiligo melanocytes from H_2_O_2_-induced damage in the context of vitiligo. Additionally, Yang et al. (50) reported that BAI protected melanocytes by fostering GPX4 expression, which markedly mitigated RSL3-induced damage. Their research also suggested GPX4 as a potential therapeutic target for BAI in the treatment of vitiligo.

### Potential of KF in enhancing melanin production

KFl, also referred to as kaempferol-3-O-glucorhamnoside, is a flavonoid compound primarily derived from the rhizomes of *Kaempferia galanga* in the *Zingiberaceae* family. It is also commonly found in a variety of fruits, vegetables, and beverages, and exhibits a range of biological activities, including anti-cancer, anti-inflammatory, antioxidant, anti-bacterial, and anti-viral properties. Xie et al. (51) verified that KF reinforced the phosphorylation of ERK1/2, boosted the synthesis of MITF, TYR, TRP1, and TRP2, and negated ROS-induced damage in TYR, TRP1, and TRP2, thereby enhancing melanin production. This study established the potential of KF to stimulate melanin synthesis in normal human melanocytes while simultaneously reducing oxidative stress. Giri et al. (52) investigated the *in vitro* therapeutic potential of harmine and KF for the management of generalized vitiligo (GV). Their research documented that KF-treated Tregs altered the proliferative capacity of CD8^+^ and CD4^+^ T cells and diminished IFN-γ production, thereby contributing to the survival and proliferation of melanocytes. Although these compounds offer promise as novel Treg-based therapies for GV, further *in vivo* studies are required to evaluate the safety and effectiveness of KF in GV treatment.

## TCM compounds as emerging therapeutic agents in SjS

SjS is a systemic autoimmune disorder characterized by exocrine gland dysfunction and mucosal surface dryness (53). Key symptoms include dry mouth and dry eyes, which may progress to systemic manifestations, involvement of internal organs, and an elevated risk of developing hematologic malignancies (54). The widely accepted pathogenesis of SjS is “autoimmune epithelitis” (55), though other research has suggested that the disease may also be influenced by hormonal and neuropeptide regulation of exocrine gland function (56). The current treatment options for primary SjS (pSS) mainly include topical tear and saliva substitutes, immunosuppressants, and systemic secretagogues. Unfortunately, these approaches are frequently ineffective, poorly tolerated, and lack robust clinical trial support, highlighting the urgent need for novel therapeutic approaches to SjS (57). Despite the challenges faced in the research and development of TCM compounds, several significant breakthroughs have been achieved over the past centuries.

### Total glucosides of paeony for immune modulation

TGP is a group of glycoside compounds extracted from *Paeonia lactiflora*, a traditional medicinal herb in the *Ranunculaceae* family. This mixture includes paeoniflorin, benzoylpaeoniflorin, hydroxy-paeoniflorin, paeoniflorin, albiflorin, and other biologically active compounds derived from the Rhizoma of *Paeonia lactiflora*, collectively known as TGP (58). Clinically, TGP is commonly used as an adjunct treatment for rheumatoid arthritis (RA) to relieve joint symptoms. Given its immunomodulatory and anti-inflammatory effects, TGP is also employed in the management of SLE and SjS. Early research by Wu et al. (59) on the effects of TGP in mitigating SjS uncovered that TGP lowered the levels of pro-inflammatory cytokines in the serum and submandibular glands of SjS patients. Furthermore, it reduced the IFN-γ/IL-4 ratio in the submandibular glands. TGP also appeared to restrict the expression of Fas and FasL, modulate Th1/Th2 immune balance and diminish apoptotic potential, thereby achieving beneficial therapeutic outcomes in the context of SjS. Liu et al. (60) further delineated the impacts of TGP on constipation and intestinal inflammation in the SjS mouse model. They noted that the application of TGP capsules alleviated the dryness and roughness of feces, with significant improvements in fecal gloss, quantity, and water content. Moreover, TGP repressed the expression of NO, NOS, IL-1β, TNF-α, NF-κB, and SP in the serum, while increasing VIP levels and upregulating SCF and c-kit mRNA expression in the colon. The study of Jin et al. (61) systematically reviewed clinical randomized controlled trials, involving 443 patients, to assess the clinical efficacy and safety of TGP in the treatment of pSS. Their data implied that the combination of TGP with immunosuppressants yielded better improvements in exocrine function and inflammatory response than immunosuppressants alone in the context of SjS. Similarly, a meta-analysis by Zhang et al. (62) concluded that TGP combined with hydroxychloroquine led to more satisfactory outcomes than the individual application of hydroxychloroquine in relieving dryness symptoms, facilitating tear and saliva production, and curtailing inflammation and immunoglobulin levels, all without notable adverse effects. This combination therapy has thus emerged as a safe and effective clinical approach for the treatment of SjS.

### RES for alleviating salivary gland dysfunction

RES, a non-flavonoid polyphenolic compound, is predominantly derived from such plants as *Reynoutria japonica* and *Vitis vinifera*. It has been well-recognized for its anti-aging, anti-cancer, and cardiovascular protective properties, making it a promising ingredient for health food products and potential pharmaceutical development. Notably, Inoue et al. (63) used non-obese diabetic (NOD) mice with impaired lacrimal gland and SG functions as an animal model for SjS. Their observations indicated that RES administration expedited saliva secretion and intensified IL-10 expression in the SGs, indicating that RES may alleviate salivary dysfunction in patients with SjS. This conclusion was further supported by Yoon et al. (64), who examined the mechanistic underpinnings of the role of RES in mitochondrial RNA-mediated cellular responses to immunogenic stress. They verified that RES preserved the integrity of tight junction complexes and aquaporin expression patterns. This observation is consistent with the earlier conclusion that RES enhances salivary dysfunction in mouse models. Nonetheless, the precise mechanism of RES in controlling mitochondrial RNA in the context of SjS remains unclear and warrants further characterization. In addition, Zhang et al. (65) substantiated that RES mitigated SG damage by modulating immune infiltration disturbances, and validated that RES was identified as the drug most closely associated with cuproptosis-related genes involved in SjS-related SG injury.

### Artemisinin for immune regulation and symptom relief

ART, an active ingredient extracted from the Chinese medicinal herb *Artemisia annua*, is extensively recognized as the most effective low-toxicity antimalarial drug (66). Moreover, ART and its derivatives have demonstrated therapeutic potential in treating a range of inflammatory and immune-mediated conditions, including RA and SLE (67). For mechanistic studies, Liao et al. (68) employed network pharmacology to clarify the potential molecular targets of ART in the treatment of pSS. Their analysis pinpointed 412 targets and 1,495 related genes, with 40 intersecting genes between ART and pSS. Further molecular docking studies and *in vivo* experiments suggested that the therapeutic action of ART in mitigating the dysfunction associated with pSS is strongly linked to the modulation of the balance between Tregs and Th17 cells. Researchers have also examined the relationship between artesunate, a derivative of ART, and its potential therapeutic effects on SjS. Zhang et al. (69) provided evidence confirming that artesunate ameliorated SjS-like symptoms in NOD/Ltj mice and curbed B cell-activating factor-induced B cell hyperactivation by modulating the TRAF6/NF-κB signaling pathway. Interestingly, Xiao et al. were among the first to observe that artesunate prominently thwarted the proliferation and differentiation of Th17 cells in both mouse and human cell cultures. Given that Th17 cells are critical drivers of the onset and progression of SjS, their findings suggest a potential therapeutic role for artesunate. Furthermore, artesunate was found to mitigate the Th17 response by expediting IRF4-mediated inhibition of glycolysis, thus alleviating symptoms related to SjS (70). Additional studies have also summarized the role of ART in the treatment of SjS.

### Lycium barbarum polysaccharide and apigenin for glandular function protection

LBP, a traditional Chinese medicinal compound extracted from *Lycium barbarum* and other fruits, has been extensively studied for its diverse therapeutic properties, particularly its immunomodulatory and anti-inflammatory effects. Although high doses of LBP have been validated to enhance immune function by stimulating T cell proliferation, Wang et al. (71) uncovered that even low doses of LBP could notably repress SG inflammation in a pSS mouse model. The study further verified that LBP preserved salivary secretion function and slowed the progression of pSS by balancing Tregs and Tfh cells and diminishing apoptosis. These promising results suggest that LBP may offer a novel therapeutic approach for treating pSS and other autoimmune diseases.

APG, a flavonoid derived from *Apium graveolens*, various fruits, legumes, and tea, has earned recognition for its pharmacological properties, including anti-cancer, anti-viral (against HIV and other viruses), MAP kinase inhibition, and anti-inflammatory effects. Unlike other flavonoids such as QUE and KF, this compound exhibits low toxicity and non-mutagenic characteristics, making it a safer alternative for therapeutic applications. Xerostomia, or dry mouth, is one of the hallmark symptoms of SjS. Wei et al. (72)focused on this symptom by investigating aquaporin-5 (AQP5), a specific water channel protein expressed in the salivary glands, as a target for treatment. They studied the mechanistic actions of several individual herbal compounds, APG, dauricine, protopine, and lentinan, as potential therapeutic agents for xerostomia and explored their effects on AQP5 transcription *in vitro*. The findings revealed that APG was the only compound to robustly induce AQP5 transcription and expression, thereby alleviating symptoms of xerostomia. Although LBP and APG showed promise in mitigating typical SjS symptoms, further research is necessary to fully elucidate their mechanisms of action.

## Opportunities for TCM compound research in other autoimmune-related skin diseases

Beyond the investigation of psoriasis, AD, vitiligo, and SjS, it is crucial to broaden the research on TCM compounds to include other autoimmune skin diseases, such as SLE, SSc, BD, and DM. SLE, a representative autoimmune disease, is characterized by multi-system involvement and the presence of numerous autoantibodies in the serum. Research on the treatment of SLE with TCM compounds has suggested that TCM can ameliorate disease severity by modulating the quantity and function of Tregs. For example, extracts from *Astragali Radix* have been delineated to diminish the proportion of Tregs, thereby curbing immunosuppressive activity and improving the clinical state of SLE patients (73). In the treatment of mild SLE, Xue et al. (74)indicated that the addition of TGP to conventional drug therapy potentiated the therapeutic effectiveness and improved immune parameters without increasing the risk of complications. *Radix Tripterygium wilfordii*, recognized for its potent anti-inflammatory, immunomodulatory, and analgesic effects, has been extensively applied in clinical settings to manage autoimmune disorders, such as SLE, RA, and Henoch-Schönlein purpura (75). SSc is characterized by fibrosis of the skin and internal organs. TCMs have shown considerable promise in modulating the immune responses of patients with SSc. Notably, *Ganoderma lucidum* polysaccharides have been observed to decrease the proportion of Tregs (73). Moreover, the combination of *Cinnamomi Ramulus*, *Glycyrrhizae Radix et Rhizoma*, *Astragali Radix*, and *Codonopsis Radix* may offer anti-inflammatory, anti-fibrotic, and vascular protective effects to alleviate symptoms related to SSc (76). However, the specific TCM compounds responsible for these effects require further clarification. BDs, including pemphigus and pemphigoid are characterized by blisters on the skin and mucous membranes. Recent advancements in the application of TCMs for BD treatment suggest that TCM compound formulations can improve immune responses in patients by modulating Treg functions (77). Nonetheless, further studies are needed to identify active ingredients responsible for these therapeutic effects. Lenabasum is a cannabinoid type 2 receptor (CB2R) reverse agonist that demonstrates anti-inflammatory effects *in vivo* and *in vitro* in dermatomyositis (DM) and is currently being investigated for therapeutic potential. In a recent publication, extensive profiling of dermatomyositis skin was performed and lenabasum was found to downregulate CD4-positive T cells, IFN-β, IFN-γ, IL-31 and pathophysiological CB_2_ receptor expression, while a similar effect was not observed in healthy skin (78).

## Discussion

Chinese medicine faces many obstacles in the global clinical application of skin diseases. Including the challenges of evidence-based medicine evidence, the lack of high-quality RCTs: despite the large number of clinical observations and case reports, the lack of high-quality rcts has not been addressed, however, large-scale, multicenter, randomized, double-blind placebo-controlled trials that meet the standards of modern evidence-based medicine are still relatively insufficient. This leads to its efficacy is difficult to be fully accepted by the international mainstream medical community. The other problem is the inherent contradiction of methodology: the core of TCM is “Treatment based on syndrome differentiation”, emphasizing the individualization of treatment, which is fundamentally in conflict with the research paradigm of modern RCT requiring standardized intervention. The last one is dominance of modern medical systems: globally, Diagnostic and treatment guidelines in dermatology are largely based on modern medical systems, with traditional Chinese medicine often used as a ‘complementary’ or ‘alternative’ therapy rather than as a first-line option, and as an alternative to traditional medicine, its application is limited.

But TCM compounds have emerged as a promising class of therapeutic agents with considerable potential in the treatment of inflammatory and autoimmune skin diseases, including psoriasis, vitiligo, and AD. Conventional therapies for these conditions typically involve anti-inflammatory agents, immunosuppressants, phototherapy, topical treatments, and biologics, but these therapies are frequently accompanied by adverse effects. Moreover, the use of biologics is still constrained by high cost, skin irritation, immune dysregulation, and a potential increased risk of carcinogenesis. TCM-derived compounds exhibit considerable therapeutic potential by targeting specific signaling pathways and repressing the proliferation and survival of skin cells, thus presenting a safer and potentially more effective alternative. Dysregulation of the PI3K/AKT/mTOR pathway has been linked to the pathophysiology of inflammatory and immune-mediated skin disorders. The PI3K/AKT pathway, in particular, plays a crucial role in the progression of several inflammatory skin conditions. Matrine, for example, has been verified to suppress the PI3K/AKT/FOXO signaling pathway and is used as an adjunctive therapy for psoriasis (79). The JAK/STAT signaling pathway is critically involved in the pathogenesis of skin inflammation and immune disorders. Evidence exists reporting that CUR prominently impacts the JAK/STAT3 signal transduction (80), and BBR represses CDC6 expression and the proliferation of human keratinocytes through orchestrating the JAK/STAT3 pathway (81), making both compounds potential therapeutic agents for psoriasis. In addition, PUR has emerged as a viable option for treating AD due to its inhibitory effects on chemokine expression by blocking the activation of STAT-1, MAPKs, and Akt (82). Clinical evidence indicates that JAK inhibitors may induce pigmentation in vitiligo (83). RES has been substantiated to upregulate SOCS1 and block the JAK/STAT pathway (84), and leonurine upregulates SOCS5 to inhibit the JAK/STAT signaling (85). Additionally, LUT has been demonstrated to augment SOCS3 expression in LPS- and IFN-γ-challenged microglia (BV-2 cells), leading to STAT1 inactivation and inflammation reduction (86). These studies highlight the growing potential of TCM-derived compounds as innovative therapeutic strategies for the treatment of various inflammatory and autoimmune skin diseases and provide new avenues for dermatological drug development. However, several challenges exist in the drug development process, including the potential for diminished or lost therapeutic properties during plant extraction and isolation. Furthermore, geographical location, climate, and cultivation practices can substantially influence the quality of TCM compounds (87). Further investigation is required to optimize the delivery methods of TCM compounds. Existing evidence suggests that topical application of TCM can alleviate skin dryness and repair the skin barrier in patients with AD (88). In the context of psoriasis, the combination of nanocarriers with natural drugs for targeted delivery improves the solubility and stability of drugs during transdermal absorption (89). It should also be noted that the therapeutic mechanisms of TCM compounds require further investigation to provide new treatment options for the prevention and management of dermatological diseases.

## Conclusion

This review systematically summarizes the current research on the therapeutic potential of TCM compounds in autoimmune-related skin diseases (**Table 1**), with the aim to offer mechanistic insights for further studies and new drug development. Compounds derived from TCMs have played a pivotal role in drug development. Their remarkable structural diversity makes them an excellent source of novel chemical entities for new drug discovery (**Table 2**). The present study presents a comprehensive analysis of autoimmune skin diseases, including psoriasis, AD, vitiligo, and SjS. The pathogenesis of these conditions is characterized by complex interactions among multiple factors (**Figure 1**), and they are frequently accompanied by comorbidities or secondary symptoms. Clinically, early diagnosis, prompt treatment, and the strategic use of combination therapies are crucial for improving patient outcomes and enhancing quality of life.

**Table 1:** Common TCM compounds and their applications in autoimmune-related skin diseases.

Compound name	Molecular formula	Skin diseases	References
Curcumin	C_21_H_20_O_6_	Psoriasis, vitiligo	(6–10) (41–44)
Boswellic acid	C_30_H_48_O_3_	Psoriasis	(11–13)
Resveratrol	C_14_H_12_O_3_	Psoriasis	(14–18)
Berberine	C_20_H_18_NO_4_	Psoriasis	(19–22)
Ginsenoside	Rh1: C_36_H_62_O_9_ Rh2: C_36_H_62_O_8_ Rg5: C_42_H_70_O_12_	AD	(28–30)
Ferulic acid	C_10_H_10_0_4_	AD	(31, 32)
Baicalein	C_15_H_10_O_2_	AD, vitiligo	(33, 34) (45–47)
Puerarin	C_21_H_20_O_9_	AD	(35)
Quercetin	C_15_H_10_O_7_	Vitiligo	(39, 40)
Kaempferol	C_15_H_10_O_6_	Vitiligo	(48, 49)
Paeoniflorin	C_23_H_28_O_11_	SjS SLE	(55–59) (71)
Resveratrol	C_14_H_12_O_3_	SjS	(60–62)
Artemisinin	C_15_H_22_O_5_	SjS	(63–67)
*Lycium barbarum* polysaccharide	C_5_H_8_O_4_	SjS	(68)
Apigenin	C_15_H_10_O_5_	SjS	(69)
Astragaloside	C_41_H_68_O_14_	SLE	(70)
*Radix Tripterygium wilfordii, Ganoderma lucidum* polysaccharide	C_13_H_8_O_6_ C_6_H_10_O_5_	SLE SSc	(72) (70)

AD, atopic dermatitis; SjS, Sjögren’s syndrome; SLE, systemic lupus erythematosus; SSc, systemic sclerosis; BD, bullous dermatosis.

**Table 2:** Structure diagram of common TCM compounds.

Compound name	Structure diagram
Curcumin	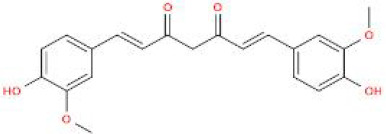
Boswellic acid	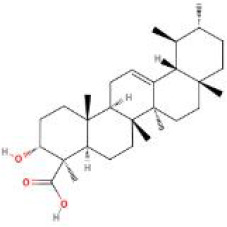
Resveratrol	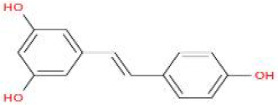
Berberine	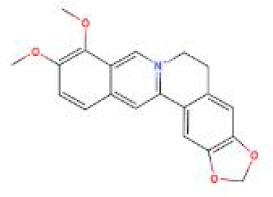
Ginsenoside	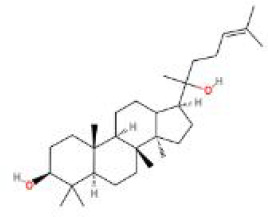
Ferulic acid	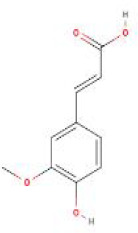
Baicalein	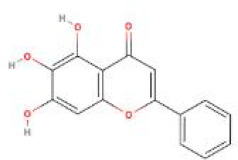
Puerarin	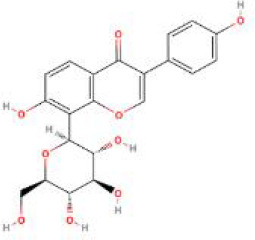
Quercetin	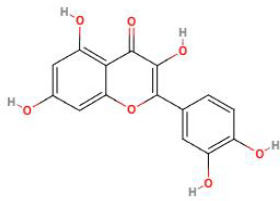
Kaempferol	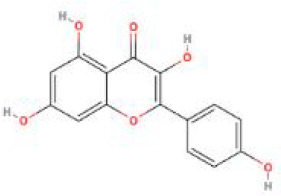
Paeoniflorin	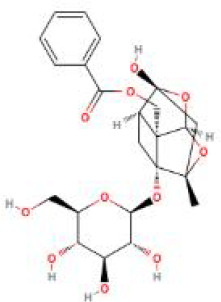
Resveratrol	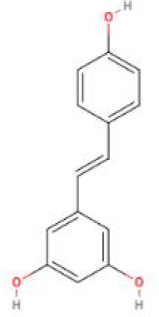
Artemisinin	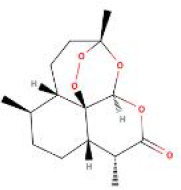
Apigenin	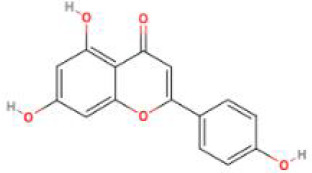
Astragaloside	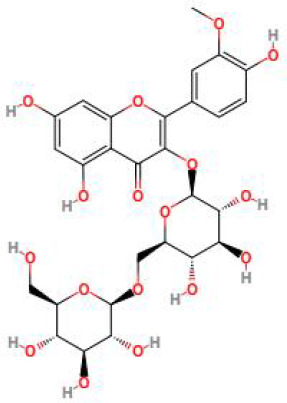

**Figure 1:**
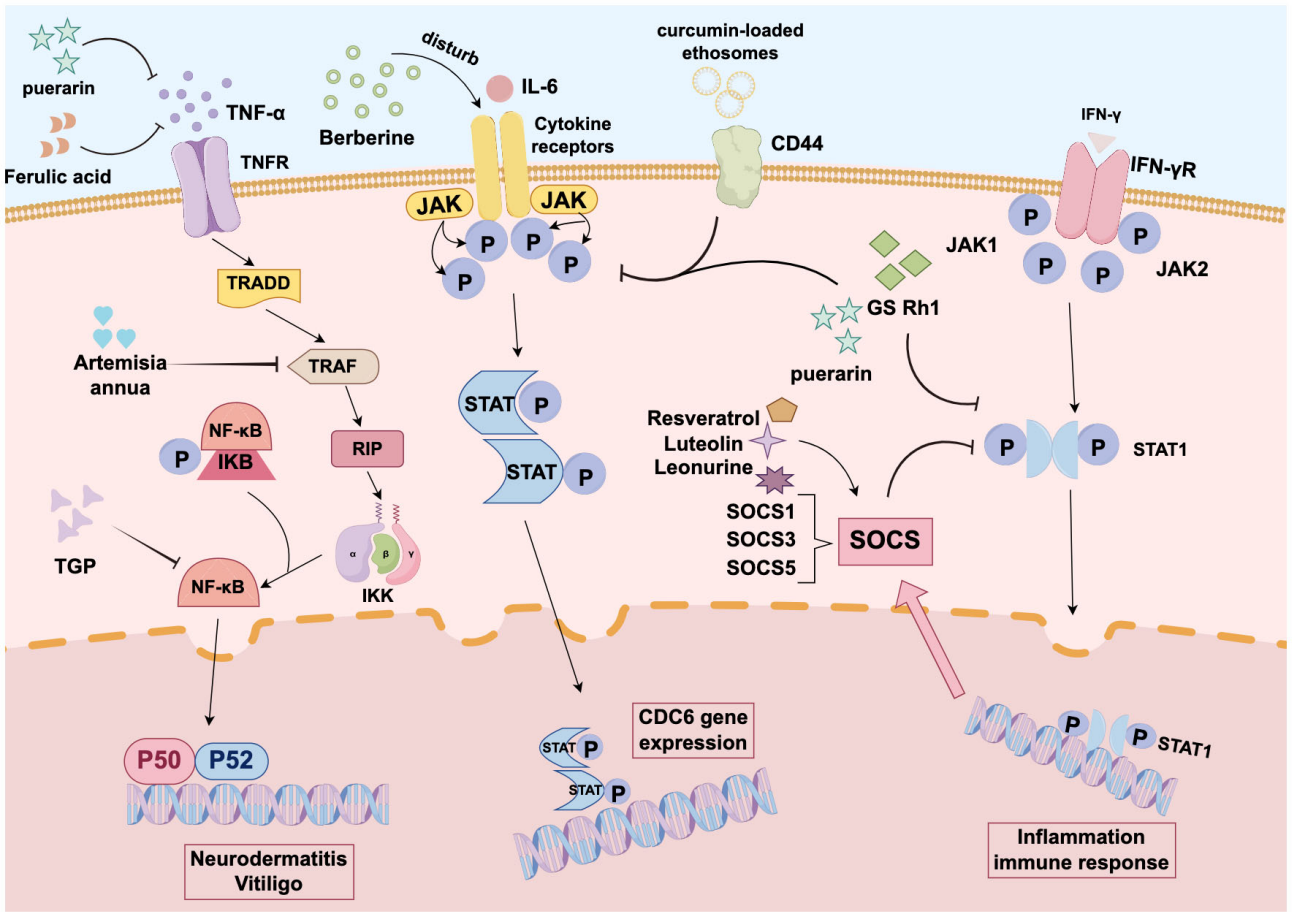
Mechanism diagram of traditional Chinese medicine components in treating autoimmune skin diseases: Curcumin is closely associated with JAK-STAT3 signaling. Berberine can also inhibit CDC6 expression and proliferation in human keratinocytes by interfering with the JAK-STAT3 pathway. Both compounds hold promise as effective therapeutic agents for psoriasis. Puerarin reduces chemokine expression by blocking the activation of pathways such as STAT1 and NF-κB, making it a viable alternative treatment for atopic dermatitis. Resveratrol upregulates SOCS1 and blocks JAK-STAT signaling. Lintepine upregulates SOCS5 to inhibit JAK-STAT signaling. Luteolin also increases IFN-γ-stimulated SOCS3 expression, thereby inactivating STAT1 signaling and attenuating inflammatory responses.
